# Radiotherapy combined with cytokine-induced killer cell therapy for liver metastasis from rectal cancer: A case report

**DOI:** 10.1097/MD.0000000000017636

**Published:** 2019-10-25

**Authors:** Yazheng Dang, Tao Qi, Hongxiang Gao, Shigao Huang

**Affiliations:** a Department of Radiation Oncology, 986 Hospital of People's Liberation Army Air Force; b Department of Radiotherapy Oncology, Chang An Hospital, Xi’an, Shaan Xi; c Cancer Centre; d Institute of Translational Medicine, Faculty of Health Sciences, University of Macau, Taipa, Macao SAR, P.R. China.

**Keywords:** cytokine-induced killer cell therapy, distant effect, liver metastasis, radiotherapy, rectal cancer

## Abstract

**Rationale:**

Colorectal cancer is the most common type of cancer leading to death; approximately 10% to 25% of rectal cancer patients present with synchronous colorectal liver metastases. However, the management of synchronous colorectal liver metastases is difficult, especially for patients unable to tolerate chemotherapy or surgery. To date, the optimum treatment of colorectal liver metastasis patients remains controversial, and the curative effect is unsatisfactory. Therefore, we established a novel therapeutic approach to treat colorectal liver metastases employing radiotherapy plus immunotherapy.

**Patient concerns:**

A 56-year-old man presented with mucous bloody defecation occurring >20 times a day and accompanied by fatigue and poor appetite. After 4 months, he was admitted to the hospital due to increased fecal blood volume.

**Diagnosis:**

Highly differentiated adenocarcinoma was diagnosed based on rectal biopsy, and abdominal computed tomography (CT) showed multiple metastatic tumors in the liver.

**Interventions:**

The patient underwent 1 cycle of chemotherapy, which was terminated owing to severe gastrointestinal reactions. Several days later, he was administered cytokine-induced killer (CIK) cell therapy plus adjuvant radiotherapy.

**Outcomes:**

Dynamic changes in the patient's tumor markers returned to normal levels, and abdominal CT and abdominal magnetic resonance imaging (MRI) revealed no metastatic liver tumors.

**Lessons:**

Sequent therapy provided a curative effect for liver metastasis in a rectal cancer patient. Radiation may have activated the body to produce distant effects, eliminating the live metastasis. CIK cell-immunotherapy and radiotherapy may have synergistic therapeutic effects and could be combined for successful treatment of liver metastasis from rectal cancer.

## 1 Introduction

Colorectal cancer is the most common cause of cancer death worldwide; approximately 10% to 25% of rectal cancer patients present with synchronous colorectal liver metastases.^[1,2]^ The optimal treatment of such patients remains controversial, and the curative effect is unsatisfactory. The only curative intervention for colorectal liver metastases has been hepatectomy^[3,4]^; however, some patients are ineligible for resection; therefore, untreated colorectal liver metastases can result in poor survival rates and life quality. One of our previous studies showed that cytokine-induced killer (CIK) cells have significant synergistic therapeutic effects against esophageal cancer.^[5]^ In the current study, we describe a case of rectal cancer successfully treated with CIK cell infusion plus adjuvant radiotherapy, following which a metastatic liver tumor disappeared. These results demonstrate that radiotherapy may activate immune cells to produce distant effects.

## 2 Case presentation

**Figure 1:**
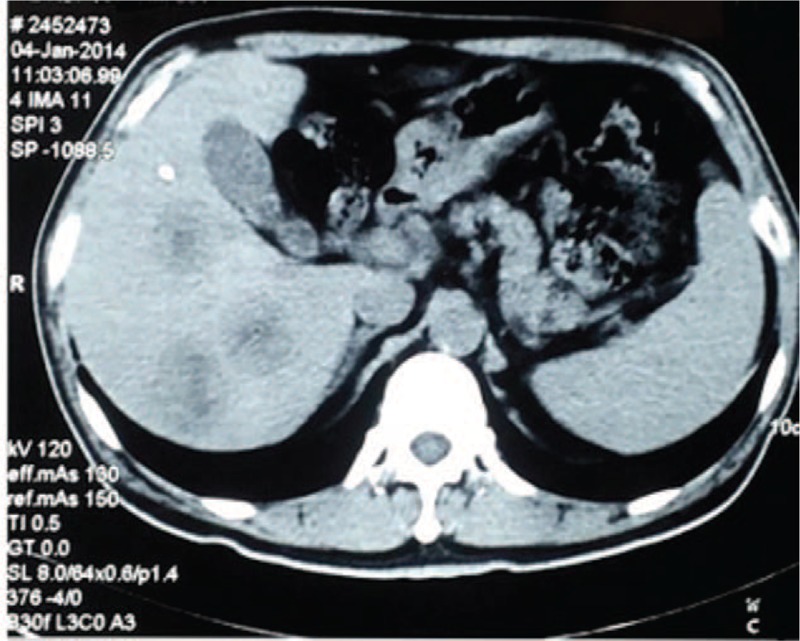
Abdominal computed tomography revealed multiple metastatic tumors in the liver before combination treatment.

**Figure 2:**
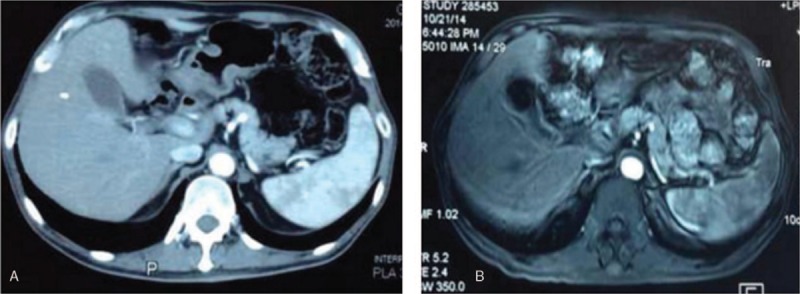
After combination treatment, abdominal computed tomography (A) and abdominal magnetic resonance imaging (B) revealed a small cyst in the right anterior lobe of the liver but no metastatic liver tumor.

**Figure 3:**
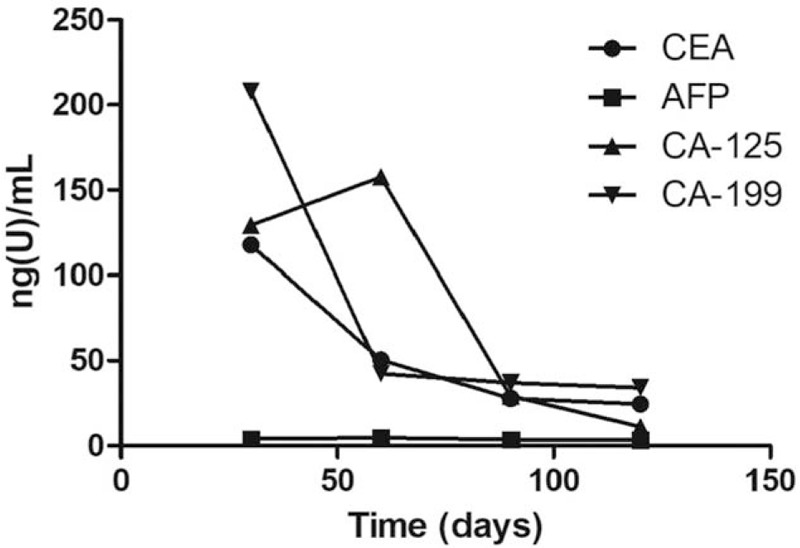
The levels of biochemical tumor markers decreased after treatment. In addition, when the patient had stable disease, the tumor marker levels were within the normal ranges, except for carcinoembryonic antigen (CEA) levels that were slightly higher than the reference range.

**Figure 4:**
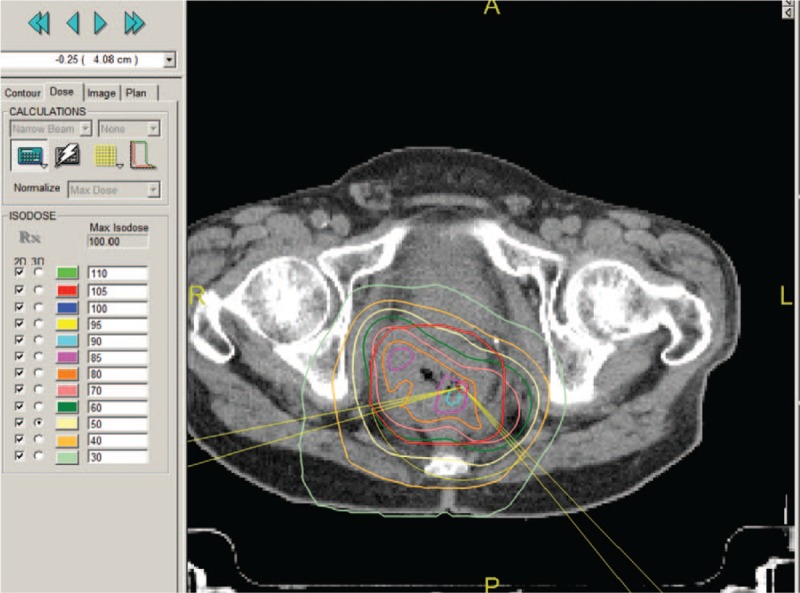
The color curve shows the radiation dose pattern, with a high dose to the tumor center and low doses to the surrounding areas. This dose was 80% of the maximum isodose (orange color curve) surrounding the tumor volume, resulting in the highest dose to the center.

A 56-year-old man visited our hospital with intermittent hematochezia for 1 year. He presented with hematochezia without any inducement, which included intermittent bloody stools, accompanied by perianal distension pain and thought to be untreated “hemorrhoids.” After 4 months, he was admitted to the hospital due to increased fecal blood volume. Rectal biopsy revealed glandular dysplasia in the rectal mucosa, indicating highly differentiated adenocarcinoma, which was at T3N2M1a stage,^[2]^ and was considered progressive disease (PD) according to response evaluation criteria in solid tumors (RECIST).^[2]^ Abdominal computed tomography (CT) revealed multiple metastatic tumors in the liver (Fig. 1). Before treatment, biochemical analysis of the patient was performed (Table 1). One cycle of FOLFOX chemotherapy was administered and then terminated due to severe gastrointestinal reactions. Several days later, episodes of mucous bloody defecation occurred >20 times a day, accompanied by fatigue and poor appetite. He was then treated with gamma knife radiotherapy for rectal lesions following the dosage fraction of 39 Gy/13 f. After radiation treatment, the frequency of defecations was reduced to >10 times, but <20 times a day, presenting as mucous bloody defecation. The tumor markers CA-125 and CA-199 had decreased sharply (Table 1). After 15 days, he was injected with allogeneic CIK cells (1 × 10^9^/250 mL, once a week for 3 weeks), and the treatment process proceeded smoothly, without the patient experiencing any special discomfort. After half a year, reexamination with abdominal CT (Fig. 2A) and magnetic resonance imaging (MRI) (Fig. 2B) was performed, and the results revealed a small cyst in the right anterior lobe of the liver; no liver metastatic tumor was found. Moreover, the patient's acute and late toxicities of gastrointestinal and hematological adverse event were decreased to be Grade 1 according to Common Terminology Criteria for Adverse Events v3.0 (CTCAE).^[11]^ In addition, dynamic changes in the patient's tumor markers had returned to normal levels as described in Fig. 3, and the dosage performance of gamma knife radiotherapy for rectal lesions is described in Fig. 4. The clinical study was approved by the Ethical Committee of the 986 Hospital of People's Liberation Army Air Force, and the participant signed an informed consent about the benefits and risks of the procedure. Patient has provided informed consent for publication of the case.

**Table 1:**
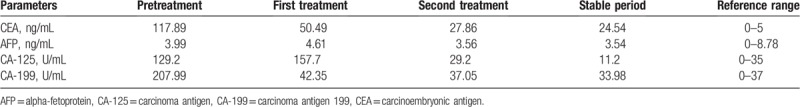
Biochemical tumor markers for patient.

## 3 Discussion

Immunotherapy for liver cancer (immune cell and gene therapies, molecular targeting, endocrine and stem cell therapies) is the fourth most commonly employed treatment, preceded only by surgery, radiotherapy, and chemotherapy.^[6]^ Because a single treatment can only eliminate a tumor visible to the naked eye, the tumor cells circulating in the blood are the key factors for early metastasis and recurrence of liver cancer; therefore, a single treatment is not satisfactory. Mesiano et al^[7]^ found that the numbers of CD3+ and CD4+ NK cells in tumor patients are significantly lower than those in healthy people. Moreover, tumor cells themselves can secrete immunosuppressive substances (such as interleukin [IL]-4 and IL-10), which can reduce the body's response to them, thus facilitating their growth and metastasis.

CIK cells, also called natural killer (NK) cells, are a subset of T lymphocytes,^[8]^ which are a group of immune effector cells featuring a mixed T- and NK-cell-like phenotype. CD3- and CD56-positive cell markers on a CIK cell's surface provide some functions of T lymphocyte and NK cell characteristics, resulting in a CIK cell's more powerful effect in killing tumor cells because other auxiliary cells and factors that play a direct anti-tumor role are not needed. CIK cells also play a certain role in malignant tumors that are not sensitive to radiation and chemotherapy drug treatments. Recently, preclinical trials and animal experiments on the application of CIK cells in the treatment of liver cancer have been widely conducted, both domestically and abroad, and a large number of studies in the literature have reported good prospects for the application of CIK cells.^[9]^ Moreover, it has been reported that normal human CIK cells have obvious proliferation potential in vitro and have significant anti-graft tumor activity.^[7,9–11]^ Presently, the primary sources of CIK cells are autologous, allogeneic, and umbilical cord blood and bone marrow derived. Use of autologous CIK cells can avoid cross infection, and CIK cells from liver cancer patients have strong cytotoxic effects on liver cancer cells, as do CIK cells from normal people; however, they cause no damage to normal liver cells. Allogeneic CIK cells, which are obtained primarily from normal people, from a wide range of sources, can increase the chance of accidental infection. The classical culture method for obtaining CIK cells is to isolate mononuclear cells after the extraction of a patient's peripheral blood, and then to add a variety of cytokines to induce 2-to-3 weeks of amplification culture, yielding a large number of CIK cells with high killing activity; these active cells are returned to the patient to play an anti-tumor role. The mechanism of CIK cells killing tumors is as follows: first, CIK cells recognize and bind tumor antigens on tumor cells through their antigen receptors, and directly kill tumor cells through cell lysis and cytotoxic molecules such as granzyme and perforin.^[12,13]^ Second, CIK cells indirectly kill tumor cells by secreting several cytokines such as interferons (IFNs) and tumor necrosis factors (TNFs).^[14,15]^

In the current study, the patient was a 56-year-old man with colorectal liver metastases, who was ineligible for surgery and unable to tolerate chemotherapy. He then underwent radiation therapy for rectal lesions plus dendritic cell (DC)-CIK cell therapy. Following treatment, the patient returned to a normal defecation pattern, his liver metastases disappeared, his abnormal stool symptoms improved, and his liver metastasis disappeared. This patient was treated by gamma knife radiotherapy with the following prescription dose: 3.0 Gy/fraction of approximately 50% of the dose curve area surrounding the tumor edge, for a total of 13 times, up to a total dose of 39 Gy. A single dose in the center area was very high, reaching approximately 4.8 Gy. This was 80% of the maximum isodose surrounding the tumor volume, which had the highest center dose. This scenario is analogous with an onion structure, having a high center dose layer and a low around dose layer (Fig. 4).

Local radiotherapy combined with immune stimulation can produce the abscopal effect, which is an immune-mediated systemic anti-tumor response.^[16–18]^ The ability to purposefully and reliably induce abscopal effects in metastatic tumors could meet many clinical needs.^[19]^ However, recently an increasing number of studies have reported that local radiotherapy can promote tumor-related antigen release and major histocompatibility complex (MHC) molecular I expression.^[17–19]^ This action is synergistic with immunotherapy in enhancing the body's anti-tumor immune response. The effect time of tumor radiation therapy, which is after 6 to 8 months, coincides with the abscopal effects time.^[18–20]^ However, there are some limitations of this case report. First, it is only one patient that acquires satisfactory clinical result through radiotherapy combined with CIK-cell therapy. Second, more patients and hospitals need to identify this clinical curative effect regarding cancer treatment. The third, but not least we should improve the immune cell activity and function to make them more precise and anti-tumor effective. In the future, more mechanism about radiotherapy inducing the tumor abscopal effects in metastatic tumors should be clarified. Moreover, it has been reported that CIK cell treatment kills tumors, but whether radiotherapy can further regulate immunity and activate certain immune factors to achieve anti-distant metastatic tumor effect has not yet been reported. Whether radiation therapy can regulate immune function in patients and activate immune factors to achieve an antitumoral goal remains an unanswered question. We will perform further research to determine appropriate immune agents to treat different types of cancer, the best total dose radiation segmentation model, and the optimal joint timing to acquire a curative effect from radiotherapy combined with immune therapy.

## 4 Conclusion

In summary, the potential of radiotherapy combined with CIK cell therapy for liver metastasis from rectal cancer should be emphasized in cancer treatment. In our case, radiotherapy first reduced the primary tumor, then immune cells caused a distant effect to eliminate the liver metastasis. We believed that this combined methodology should be considered as a potentially effective and safe therapeutic regimen for treating, not only the cancers reported herein, but also other cancers. Taken together, CIK cell-immunotherapy and radiotherapy may have synergistic therapeutic effects and could be combined for successful treatment of liver metastasis from rectal cancer.

## Author contributions

**Conceptualization:** Yazheng Dang, Hongxiang Gao, Shigao Huang.

**Data curation:** Yazheng Dang, Tao Qi, Shigao Huang.

**Investigation:** Yazheng Dang, Tao Qi, Shigao Huang.

**Methodology:** Tao Qi, Shigao Huang.

**Resources:** Yazheng Dang, Shigao Huang.

**Supervision:** Shigao Huang.

**Writing – original draft:** Shigao Huang.

**Writing – review & editing:** Yazheng Dang, Tao Qi, Hongxiang Gao, Shigao Huang.
